# Chordoid Glioma of the Third Ventricle: A Case Report and a Treatment Strategy to This Rare Tumor

**DOI:** 10.3389/fonc.2020.00502

**Published:** 2020-04-09

**Authors:** Xiao Chen, Benyan Zhang, Sijian Pan, Qingfang Sun, Liuguan Bian

**Affiliations:** Department of Neurosurgery/Pathology, Ruijin Hospital, School of Medicine, Shanghai Jiao Tong University, Shanghai, China

**Keywords:** chordoid, chordoid glioma, glioma, third ventricle, Gamma Knife radiosurgery, histopathology

## Abstract

Chordoid glioma (CG) of the third ventricle is a rare type of brain tumor. Here, we present a case, review of the literature and proposed a treatment strategy for this rare tumor. Here, A 33-years-old woman presented with the menstrual disorder and progressive obesity. Magnetic resonance imaging showed a large irregularly circular tumor in the third ventricle. The tumor was subtotally resected by microsurgery via the right modified port approach. Immunohistochemical staining was positive for glial fibrillary acidic protein (GFAP), Vimentin and transcription termination factor-1 (TTF-1), and the Ki-67 proliferation index was low (5%), which indicating CG. Residual tumor decreased after treated by Gamma Knife radiosurgery (GKRS) with a dose of 15 Gy. During 30 months of follow-up, the tumor did not recur, and the patient suffered no complications. The diagnosis of CG requires a combination of clinical presentation, neuroimaging, and pathology. The ideal therapy is gross total resection (GTR) of the tumor. However, GTR is usually difficult and carries a high risk of postoperative complications because of the tumor location. This case indicates that planed subtotal resection followed by GKRS with a proper marginal dose could be a good treatment strategy for CG.

## Background

Chordoid glioma (CG) is a rare central nervous system neoplasm, typically arising from the anterior wall or roof of the third ventricle. CG was first reported by Brat et al. (1) as a new distinct histopathological tumor entity, and the World Health Organization (WHO) 2007 classified it as a grade II neoplasm (2). No consensus has been reached on the histogenesis of the tumor, but a common hypothesis supports a glial origin, particularly from the ependymal cells. Other hypotheses favor divergent neuronal, glial, circumventricular, and subcommissural differentiation (3–5). Clinical, radiological, and clinicopathological features of CGs are usually pleomorphic and can mimic those of other types of lesions (4, 6). We report the case of a patient with CG presenting with progressive obesity and menstrual disturbance and review the diagnostic criteria and management strategies for this rare neural tumor.

## Case Presentation

A 33-years-old woman presented with 5 years of progressive obesity and 2 years of menstrual disturbance. Her menarche age was 15, and her cycle was 35 days with a 5–7 days menstrual period. The patient appeared overweight when she was 4 weeks pregnant in 2009. Her pregnancy ended with a cesarean section at 33 weeks, but her weight progressively increased from 50 to 93 kg. She lost 15 kg in weight after controlling her diet, but her weight increased to 100 kg after she resumed a normal diet. In 2013, she began to experience menstrual disturbances, including hypomenorrhea, delayed menorrhea, and amenorrhea. At this point, she visited the local hospital. Estradiol was administered to regularize the menstrual cycle, although without estradiol treatment she experienced abnormal cycles. The patient was then referred to our hospital.

**Figure 1:**
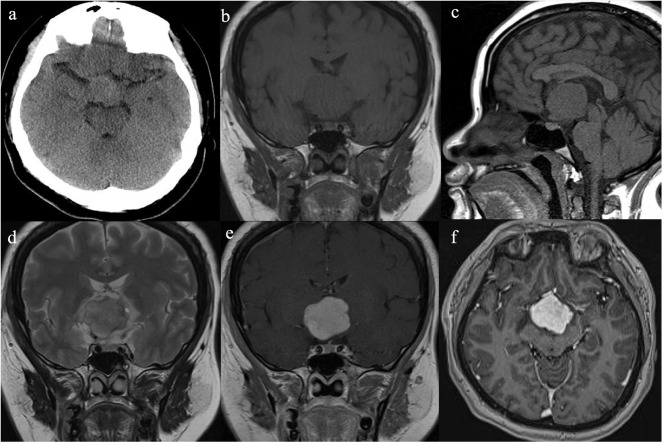
Radiological evaluation of CG preoperatively. A none-contrast CT scan showed a slightly hyper-dense mass located at suprasellar and the third ventricle **(a)**. The tumor was slightly hypo-intense on Coronal and Sagittal T1WI **(b,c)** and slightly hyper-intense on Coronal T2WI **(d)**. Post-contrast (gadolinium-enhanced) Coronal and Axial MRI **(e,f)** show prominent homogeneous enhancement.

She had no history of smoking and alcohol consumption and any significant past medical history. She also had no family history of cancer. She had normal moods and cognition and no obvious memory disturbance. Visual problems and galactorrhea were excluded. The general physical and specific neurological examinations were unremarkable. Her weight and height were 105 kg and 156 cm, respectively, and she had a body mass index of 43.15 kg/m^2^ and blood pressure of 138/88 mmHg. Hormonal, serological, and urinary examinations indicated no abnormality; select hormone levels are shown in Table 1. Chest radiography indicated no suspected lung mass or pathology. Brain non-contrast computed tomography showed a well-circumscribed, irregularly ovoid tumor in the third ventricle, which was slightly hyperdense (Figure 1a). Magnetic resonance imaging (MRI) showed a well-defined ovoid mass measuring 4.5 × 3.3 × 4.1 cm. The tumor demonstrated intermediate signal intensity on T1-weighted images (Figures 1b,c) and slightly high and high signal intensity on T2-weighted images (Figure 1d). Post-contrast enhancement images indicated prominent homogeneous enhancement (Figures 1e,f). The lesion was separated from the pituitary. Hydrocephalus was not detected. Clinical symptoms and radiological data were discussed at a multidisciplinary team meeting. The tumor was considered to have arisen from the third ventricle and was therefore unlikely to be either a craniopharyngioma or a pituitary adenoma. After discussions with the patient and her family, they opted for conservative treatment, and we decided on subtotal resection (STR).

**Table 1:** Laboratory hormone levels preoperatively and postoperatively.

	**Pre-op**	**Post-op**	
	**2015/10/24**	**2015/11/11**	**2015/11/30**
T3 (0.89–2.44 nmol/L)	1.84	1.65	1.46
T4 (62.67–150.84 nmol/L)	111.44	116.34	58.43
FT3 (2.63–5.7 pmol/L)	4.72	4.08	3.69
FT4 (9.01–19.04 pmol/L)	15.06	16.05	10.18
TSH (0.35–4.94 uIU/mL)	0.9054	0.2237	0.1501
IGF-1 (75–212 ng/mL)	78		
GH (0.010–3.607 ng/mL)		0.158	
ACTH (7.0–65.0 pg/mL)	57.85	5.8	
Serum cortisol 8 a.m. (6.7–22.6 ug/dl)	18.33	1.6	2.72
Serum cortisol 4 p.m.	3.81	23.68	
Serum cortisol 0 a.m.	3.38	3.37	
Urinary free cortisol (21–111 Ug/24 h)	86.8/1.4L	2280.38/3.8L	1498.35
LH (1.80–11.78 mIU/mL)	2.86	0.72	
FSH (3.0–8.1 mIU/mL)	3.63	1.36	
PRL (3.46–19.40 mIU/mL)	5.85	11.03	
E2 (21–251 pg/mL)	49	54	
*P* (<0.1–0.3 ng/mL)	0.11	1.4	
Sodium (135–145 mmol/L)	137	158	141
Potassium (3.5–5.0 mmol/L)	4.16	4.08	3.37
Chlorine (90–110 mmol/L)	98	116	104

The surgery was performed via the right modified transpterional port approach on November 10, 2015. After opening of the sylvian cistern, carotid cistern, and optic chiasm cistern, a solid tumor was observed at the anterior of the third ventricle and the posterior of the optic chiasma. The tumor was subtotally resected, and the resected specimen measured ~3.5 × 3.0 × 3.3 cm.

**Figure 2:**
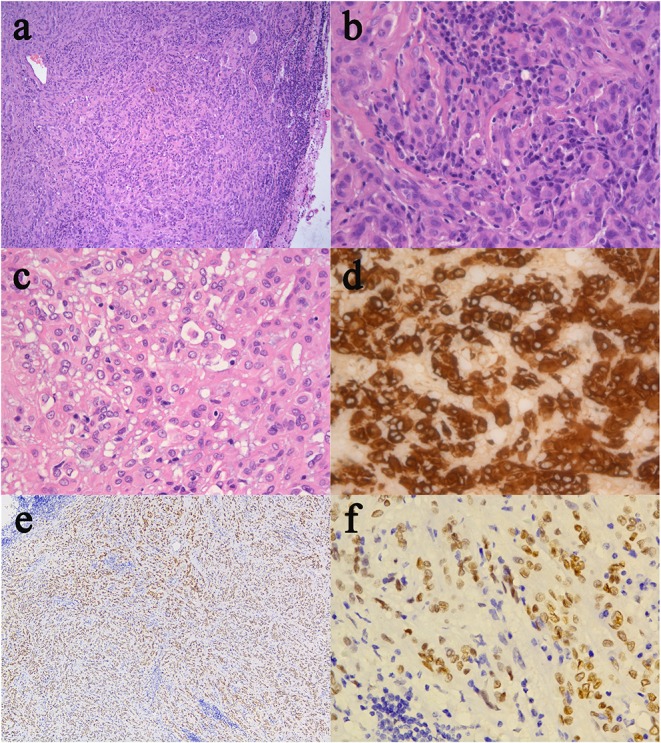
Histological and immunohistochemical features of CG. **(a)** Clusters and cords of epithelioid tumor cells are surrounded by variable mucinous stroma. A small number of normal epithelial cells are at the edge of the pathological section. There is a disorganized boundary between them. **(b)** Neoplastic cells disposed of in cords lying in a myxoid matrix with scattered lymphocytes and plasmocytes. **(c)** Epithelioid elements immersed in abundant lymphocytes and plasmocytes stroma. **(d)** The immunostained slide showed tumor cells were positive for GFAP. **(e,f)** Furthermore, tumor cells were diffusely positive for TTF-1. (Original magnifications: **(a)** 100×; **(b–d)** 400×; **(e)** 50×; **(f)** 400×).

The resected specimen was moderately pleomorphic with oval/elongated nuclei, evenly distributed, and locally epithelioid, with infiltration of lymphocytes and plasma cells. Immunohistochemical staining was positive for glial fibrillary acidic protein (GFAP), vimentin and transcription termination factor-1 (TTF-1) and negative for S-100, CK, SYN, CgA, CD56, epithelial membrane antigen (EMA), Cam5.2, D2-40, LH, ACTH, PRL, FSH, TSH, and GH (Figure 2). The Ki-67 proliferation index was low (5%), and the tumor had low-grade features consistent with CG. According to these findings, it was diagnosed as CG of the third ventricle (WHO grade II).

**Figure 3:**
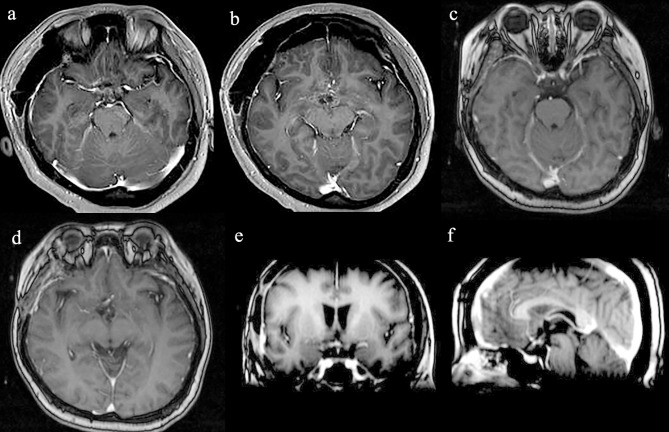
Radiological evaluation of CG postoperatively. Post-contrast MRI images of the residual tumor 1 month **(a,b)**. The size of the residual tumor decreased following GKRS **(c–f)**.

The patient recovered well after surgery. However, she developed postoperative electrolyte disturbance with alternating diabetes insipidus and syndrome of inappropriate antidiuretic hormone secretion. There were no endocrine defects apart from decreased cortisol and ACTH levels in the plasma (Table 1). After 20 days, the electrolyte imbalance subsided. However, MRI 1 month postoperatively showed residual tumor under the third ventricle measuring 1.07 × 0.81 × 0.78 cm (Figures 3a,b). Adjuvant GKRS of the residual tumor with a dose of 15 Gy was advocated by the multidisciplinary team in our hospital on March 22, 2016. The size of the residual tumor decreased following radiosurgery (Figures 3c–f). During 30 months of follow-up, the tumor did not recur, and the patient suffered no complications.

Informed consent was obtained from the patient and her relatives to publish her case.

## Discussion

CG of the third ventricle is a rare, non-invasive, slow-growing, neoplasm of the central nervous system that usually arises from the anterior part of the third ventricle. CG is frequently adherent to the hypothalamus and variably extends to the suprasellar region and lateral ventricles (1, 2). It currently has no definite risk factors or syndromic neurological symptoms. The early clinical presentation of CG varies, from being asymptomatic with incidental detection to presenting significant symptoms, depending on tumor location and size (6). Additionally, presenting with multiple symptoms rather than one presenting feature is common (6–8). Our patient presented with endocrine disorders, which may have been due to the tumor's tendency to distort the hypothalamic structure, causing effects similar to those of a pituitary adenoma (8, 9). Therefore, clinical symptoms need to be associated with imaging findings.

Enhanced MRI of the brain with gadolinium is reported to be the best diagnostic imaging tool for the evaluation of CG. CG is typically a well-circumscribed, round or oval-shaped tumor. CGs have isointensity on T1-weighted images and slight hyperintensity on T2-weighted images (10–12). After gadolinium injection, they have strong homogenous enhancement, and cystic changes and necrosis may be present, but calcifications are rare (8, 11–13). Our patient's imaging findings were consistent with those previouly described, and the tumor was separated from the pituitary, probably arising from the third ventricle, so we thought this lesion might be CG rather than craniopharyngioma or pituitary adenoma. Therefore, surgery was performed via the right modified transpterional port approach. MRI not only helps to diagnose CG but also suggests the most suitable surgical approach.

Microscopically, the tumor was moderately pleomorphic with evenly distributed oval/elongated nuclei, and epithelioid cells in some areas, with abundant infiltration of lymphocytes and plasma cells. The tumor in our case was positive for GFAP and vimentin. In some cases, immunohistochemical analysis showed that tumor cells were diffusely positive for CD34 and focally positive for EMA, pan-cytokeratin, and S-100 protein (3, 9, 14–16). CG usually has low mitotic activity, with Ki-67 values typically below 5%, as observed in our case (7, 15, 17). The tumor in our case was also TTF-1 positive. TTF-1, which is not only involved in the development of the ventral forebrain but has also been demonstrated in suprasellar tumors arising from pituicytes, has been reported to be consistently expressed in CGs (4, 16, 17). This might explain why patients have symptoms similar to those of pituitary adenoma, such as menstrual disturbance and progressive obesity. Further, CGs and pituitary adenoma might be a spectrum of tumors originating from the basal forebrain and share similar characteristics (4, 16, 18).

Although CGs are adherent to the adjacent structures, they do not invade the parenchyma. Despite being a low-grade tumor, the prognosis is usually poor because of the location in the deep structure of the brain and the difficulty in performing GTR without causing severe complications (9, 13, 19). However, partial resection of the tumor is associated with high recurrence rates (9, 19, 20). GTR has been shown to be associated with longer recurrence-free survival and better quality of life than STR, although some studies have found that mortality and morbidity rates are much higher following GTR (29 and 67%, respectively) than STR (14 and 50%, respectively) (9, 20, 21). Better tumor control would be achieved with more aggressive resection, but early surgical complications might unavoidably negate the benefit of a more aggressive approach (9). STR was performed in our case via the right modified transpterional port approach, which allowed an adequate exposure of the tumor region with a less invasive parenchymal approach (7). The operation was successful without serious complications. A transient electrolyte disorder occurred postoperatively, which improved after treatment. Adjuvant Gamma Knife radiosurgery (GKRS) of the residual tumor was then advocated. There is no current standard for radiography treatment for CG as there are few such reported cases. GKRS was first used for a residual tumor after STR in 1999, but the radiation dose and outcome were not described (14). In subsequent studies, the radiation dose varied from a marginal dose of 10.5–11 Gy to a maximal dose of 21–22 Gy, which was able to stop tumor progression (9, 10, 18–20). Referring to the previous literature (9, 10, 20), we chose a radiation dose of 15 Gy. There were no postoperative radiotherapy complications, and the size of the residual tumor decreased following radiosurgery. During the 30-months follow-up, the tumor did not recur. With the experience of this case, we propose that in patients in whom GTR is estimated to be too risky, planned STR followed by GKRS with a proper marginal dose could be a safe, effective, and minimally invasive treatment strategy for CG. We recommend that GKRS could be commenced with a low radiation dose of 10 Gy, gradually increasing the dose to 15 Gy and further to 20 Gy if the tumors don't shrink significantly. Further data is necessary to determine the optimal modality and dose of radiotherapy in the treatment of these rare tumors.

CGs are a rare subtype of low-grade glioma typically located within the third ventricle. Despite its generally slow growth, treatment-related morbidity of CGs is very high. The diagnosis of CGs requires a combination of clinical presentation, neuroimaging, and pathology. Surgical intervention remains critical in the management of CG, although GTR may not always be achievable when the tumor is close to sensitive neurovascular structures. Therefore, treatment should be tailored to individual patients. Our case suggests that planned STR followed by GKRS with a proper marginal dose could be a good treatment strategy for CG, improving tumor control or even causing regression.

However, because of the rarity of this type of tumor, we have only treated this one patient. The surgical approach, treatment strategy, and dose of GKRS were chosen on the basis of published articles. We present this case in detail and confirm the effectiveness of this treatment strategy to provide guidance for such cases in the future.

## Data Availability Statement

The original contributions presented in the study are included in the article, further inquiries can be directed to the corresponding author.

## Ethics Statement

Written informed consent was obtained from the patient for the publication of this case report.

## Author Contributions

SP, QS, and LB have been involved in the operation and management of the patient. XC and LB designed the report. XC and BZ reviewed the literature and drafted the article. BZ prepared histology figures and provided immunohistochemical analysis.

### Conflict of Interest

The authors declare that the research was conducted in the absence of any commercial or financial relationships that could be construed as a potential conflict of interest.
